# Opportunities and challenges of modern mobile housing systems for laying hen welfare - an observational study

**DOI:** 10.1016/j.psj.2026.106992

**Published:** 2026-04-22

**Authors:** K. Dorkewitz, C. Keppler, D. Gieseke, S. Horbach, U. Knierim

**Affiliations:** a Farm Animal Behavior and Husbandry Section, University of Kassel, Nordbahnhofstraße 1a, 37213 Witzenhausen, Germany; b The Hesse Department of Agricultural Affairs (Landesbetrieb Landwirtschaft Hessen, LLH), Kölnische Straße 48-50, 34117 Kassel, Germany

**Keywords:** Mobile housing, Laying hen, Welfare, Housing system, Free-range

## Abstract

A variety of commercial mobile houses for laying hens are emerging as innovative housing systems. However, only limited research on their potential for animal welfare is currently available. In this study, we compared welfare outcomes between different categories of mobile houses and with stationary systems, and aimed to identify welfare-relevant challenges associated with managing the mobile housing systems. Over a period of two years, animal-based welfare indicators (MTool©) were repeatedly assessed on 42 farms with 48 mobile houses across four size and mobility categories: wheel-based small, medium, large, and skid-based, with 200 to 2,059 hens/house. For the comparison with stationary housing, repeated MTool©-assessments from five farms with and four farms without free-range access with 2,250 to 6,000 hens/flock were used. Prevalences of plumage damage and injuries were significantly lower in mobile housing, while keel bone damage was similarly prevalent across all housing systems. The extent of footpad dermatitis was unaffected by mobile versus stationary systems. More hens had comb and wattle injuries in mobile houses, which warrants further investigation into the influences on agonistic pecking. Among others, adequate feeding space per hen must be guaranteed within the mobile house, even if this entails additional manual labor. Further key challenges of mobile housing include the protection from predators, enrichment when free-range access must be restricted temporarily due to avian influenza, climate control at all times, and optimal relocation frequency of the mobile units. Among the four mobile housing categories, relatively few differences were found. They concerned prevalences of pale combs, footpad dermatitis and slight differences in plumage soiling at the back, possibly reflecting differences in free-range accessibility, perch and litter condition and manure collection. The mostly large range in prevalences of welfare issues between flocks suggest that appropriate management is more important than mobile housing categories which all have the potential to provide for good laying hen welfare.

## Introduction

Mobile poultry farming was already adopted in the early 19th century, when small mobile poultry houses were used to utilize spent grain directly in harvested grain fields. Later they were largely replaced by more intensive systems, but since the 2000s, the number of mobile poultry houses has again considerably increased in Germany (van der Linde, 2022). This resurgence began within organic poultry farming, addressing issues of stationary free-range systems with overuse of the range, nutrient leaching, and accumulation of pathogens near the barn (Giersberg et al., 2017; van der Linde, 2022). Mobile housing is now also widely employed on non-organic farms (van der Linde, 2022) and is generally in use year-round. Van der Linde (2023) reports over 3 million places for poultry in mobile houses in Germany across more than 3,000 farms. Of these, 98% are used for egg production and 2% for meat production, representing around 2% of Germany’s total chicken population (BMEL, 2023). Around 10% of these places are located in self-built structures, but approximately 20 companies now offer various modern types of mobile poultry houses on the German market. Mobile houses are described and considered in the Scientific Opinion on the welfare of laying hens on farm (EFSA AHAW Panel et al., 2023), but no figures on their use in European countries are provided. In North America, the term “pastured poultry” was coined by Salatin (1996) to describe a production system that differs from German mobile housing, in which small floorless pens are moved once or twice daily across pasture.

Mobile systems facilitate frequent rotation between outside areas, promoting good vegetation cover across the range. This might reduce infection pressure, provide attractive supplementary feed and encourage the performance of a broad spectrum of natural behaviors (Knierim, 2006; Campbell et al., 2021). Therefore, mobile housing systems may meet increased consumer interest in animal-friendly husbandry systems (European Commission, 2007; Clark et al., 2016) and consumers' desire that animals used for food production are visible (van der Linde and Pieper, 2018).

Mobile houses must comply with the same legal welfare standards as all other housing systems, for example stocking densities may not exceed 9 hens per m² usable floor area (EC-Council Directive, 1999), or 6 hens per m² in organic systems (EU-Commission Implementing Regulation, 2020). However, their house design must also take mobility requirements into account. For example, this applies to the shape of the house, limitations in automation, and food and water storage. The mobile houses on the market meet these requirements in different ways. They can house from about 200 to 2,500 hens and vary in their levels of mobility, autonomy, and technological sophistication. Smaller houses, in particular, can be operated autonomously through the use of solar panels, batteries, feed silo and water tanks. All mobile houses can be moved across the free-range either on wheels or skids. Skid-based poultry houses are available both with and without a floor plate, whereas the wheel-based types always include a floor plate. The littered area is either integrated or can be connected to the mobile house. Interior equipment ranges from simple single-tier floor housing to multi-level aviaries, with the level of technological sophistication usually increasing with the size of the house. The different solutions may imply different challenges for the management of the birds, but also for the birds themselves. An overview of this variation in welfare relevant challenges and the resulting welfare outcomes for mobile housing is currently lacking, as mentioned by Knierim (2006) and Campbell et al. (2021).

Therefore, the aim of this observational study was to explore the specific welfare-relevant challenges associated with managing different commercial mobile housing systems for laying hens as well as the welfare outcomes both with reference to different categories of mobile houses and in comparison to stationary systems.

## Materials and methods

### Ethical approval

The birds in this study were reared and kept for production for human consumption according to national law and guidelines, and not for experimental purposes. They were only monitored by non-invasive observation and gentle palpation during routine fixation procedures. Therefore, this study was not classified as experimental procedure according to Directive 2010/63/EU and German law, and ethical approval was waived by the competent authority Regional Council of Kassel (Regierungspräsidium Kassel; RPKS - 23-19 c 16/15-2018/5).

### Farms and birds

In total, 42 laying hen farms (22 conventional, 20 organic) with 48 mobile houses **(MH)** participated voluntarily in the study. They were recruited through advertisements in agricultural magazines, recommendations from agricultural advisors, online searches, and enquiries to mobile house manufacturers. Farms had operated mobile housing systems for an average of 4.6 years (range: 1 - 20 years) and were distributed all over Germany, but with a large proportion in central Germany. It was a convenience sample. However, the aim of the acquisition was to achieve an equal distribution of the different types of commercial mobile houses across four categories which were based on size and mobility: ”wheel-based small” (**WS**, ≤ 350 hen places, n = 13), “wheel-based medium” (**WM**, 450 - 700 hen places, n = 12), “wheel-based large” (**WL**, 1,000 - 2,050 hen places, n = 11), and “skid-based” (**SB**, 200 - 2,000 hen places, n = 11). The non-contiguous ranges reflect distinct, commercially available types of modern mobile houses rather than a continuous size scale. In total, 12 different types of commercial mobile houses from six manufacturers were included, with each type present on at least two farms. All mobile housing farms provided access to free-range areas, unless this was restricted due to highly pathogenic avian influenza (**HPAI**). Selection criteria did not include the genetic background of the hens. Most farms kept brown hybrids, a few had mixed flocks of brown and white hybrids, and some kept alternative breeds (Table 1). All hens had intact beaks.

**Table 1:** Description of the four categories of mobile housing with 12 different housing types and two categories of stationary systems examined.

Housing	No. of farms	No. of houses	No. of types of mobile houses	No. of flocks	No. of visits	Flock size Mean ± SD (Min-Max)	Flock age in weeks Mean ± SD (Min-Max)	Production system		Housing system			Genetic origin		
								Con-ventional	Orga-nic	Aviary	Floor	Brown	White	Mixed brown & white	Other origin^2^
WS	11	13	3	24	46	265.3 ± 47.1 (217 - 350)	50.6 ± 21.6 (22.1 - 120.1)	4	9	0	13	76.1%	0.0%	8.7%	15.2%
WM	11	12	3	19	40	565.3 ± 108.1 (300 - 665)	50.6 ± 20.9 (21.6 - 106.9)	8	4	12	0	85.0%	0.0%	15.0%	0.0%
WL	11	11	2	22	44	1,616.0 ± 356.4 (587 - 2,059)	50.8 ± 23.2 (22.6 - 113.0)	5	6	11	0	90.9%	0.0%	0.0%	9.1%
SB	11	12	4	21	40	1,056.9 ± 500.1 (200 - 1,948)	49.7 ± 22.7 (19.9 - 95.0)	8	4	6	6	70.0%	0.0%	10.0%	20.0%

MH	42^1^	48	-	86	170	871.7 ± 606.7 (200 - 2,059)	50.4 ± 22.1 (19.9 - 120.1)	25	23	29	19	80.6%	0.0%	8.2%	11.2%
FR	5	5	-	18	54	3,600 ± 1,442.7 (2,250 - 6,000)	53.2 ± 20.5 (22.9 - 88.9)	2	3	4	1	100%	0.0%	0.0%	0.0%
NR	4	4	-	18	62	5,170 ± 1,032.9 (3,400 - 6,000)	48.5 ± 19.8 (18.1 - 89.3)	4	0	4	0	24.2%	72.6%	3.2%	0.0%

WS: wheel-based small (217 - 350 hens); WM: wheel-based medium (300 - 665 hens); WL: wheel-based large (587 - 2,059 hens); SB: skid-based (200 - 1,984 hens).

MH: mobile housing; FR: stationary with free-range access; NR: stationary without free-range access.

1 Two farms with one house in two categories included.

2 Crème hybrids, crosses: ÖTZ Coffee and Cream, alternative breeds: green layers and Marans

Data on animal-based welfare indicators from in total 18 laying hen flocks from five stationary farms with free-range access (**FR**) and 18 flocks from four stationary farms without free-range access (**NR**) were collected in the framework of another project ("Demonstration farms for animal welfare"; Table 1). The project’s primary objective was to improve the housing conditions for non-beak-trimmed laying hens to reduce feather pecking and cannibalism. Farms could apply for participation, and selection was based on criteria related to housing conditions, farm managers' commitment, and economic viability. Most of the NR farms kept white hybrids (72.6%), while brown hybrids (24.2%) and mixed flocks (3.2%) were less common. In contrast, all FR farms kept brown hybrids in accordance with German market demands. Flock sizes ranged from 2,250 to 6,000 hens (Table 1).

### Data recording

From February 2022 to January 2024, data from 86 flocks in mobile houses were obtained during 88 farm visits within the vegetation period (April to September) and 82 visits outside of it (October to March). The aim was to visit each farm four times at approximately six-month intervals; however, this was not possible in three cases due to HPAI. Where possible, houses with young flocks were selected at the start of the study. Due to varying lengths of the production periods and avian influenza outbreaks, the number of farm visits of the same flock varied: seven flocks were assessed four times, 16 flocks three times, 31 flocks twice, and 32 flocks once. Molting was performed in ten flocks (11.6%). These flocks were not assessed during molting but at least one month after completion of the molting program (mean: 3 months, range: 1 to 5 months). In Germany, no complete removal of feed, water or light is allowed, but usually light is reduced, free-range access restricted and diet changed to induce molting.

Overall, 14% of the flocks were subject to HPAI restrictions at some point during the study period. Three of these MH records were excluded because the respective flocks had never had access to free-range areas prior to the visit. However, flocks that had access to free-range areas earlier in life and again at the time of the visit were included in the analysis.

Data collection sheets were pilot tested on two farms not participating in the project and afterwards modified to their final forms. At each farm visit data were recorded on animal-based welfare indicators, current management practices and housing conditions, and a pooled fecal sample, consisting of 20 fresh droppings, was collected for endoparasitological analysis. Welfare indicators were assessed in 50 laying hens per flock using the MTool© (Keppler and Knierim, 2020b; Table 2). The hens were randomly captured across different areas of the floor, elevated levels, and, where possible, the covered veranda and free-range. Hens of mixed genetic origins were selected according to their proportion in the group. Inter-assessor agreement between two assessors and a trainer (silver standard) was evaluated using the Prevalence-Adjusted Bias-Adjusted Kappa (PABAK) prior to and between data collection phases. Agreement for all indicators was good to very good (PABAK > 0.60 or > 0.80), in single cases acceptable (PABAK > 0.40; Mean = 0.85, SD = 0.12, Min. - Max. = 0.58 - 1.00; Table 2).

**Table 2:** Definition of the animal-based welfare indicators (MTool©; Keppler and Knierim, 2020b, slightly amended) and results (mean) of inter-assessor reliability tests (Prevalence-Adjusted Bias-Adjusted Kappa values: PABAK) between silver standard and two assessors.

Animal-based indicator	Score 1	Score 2	PABAK mean	N (birds)
Beak alterations	Spiky, sharp, overlong	Torn, broken off	0.96	184
Eye alterations	Swollen	Cloudy	0.96	184
Respiratory infection	Clear audible aggravatedly breathing sound without additional sounds	Stertorous breathing	1.00	164
Pale comb	Moderate pale	Very pale	0.86	164
Bluish comb	Bluish areas ≤ 1/3 of the comb	Bluish areas > 1/3 of the comb	0.95	164
Comb & wattle injuries	3 or more small injuries ≤ 2 mm	At least one injury > 2 mm	0.67	254
Crop alterations	Enlarged crop with water retention, soft	Enlarged crop, rigid or with palpable knot, mostly with additional water retention	1.00	164

Plumage damage:	Damaged feathers (deformed or broken off) or one featherless area < 5 cm	At least one featherless area >5 cm		
- Neck			0.66	289
- Back			0.88	164
- Belly			0.88	164
Injuries:	Less than 3 small injuries	3 or more small injuries or one injury > 1 cm		
- Back			0.77	219
- Belly			0.62	218
Feces soiling:	Feces spots visible (discoloration of plumage)	Feces soiling with adhesion, mostly extensive (at least 3 feathers stuck together)		
- Back			0.93	164
- Belly			0.76	164
Inflammation belly	Reddening without discharge	Discharge, mostly with reddening	0.82	164
Cloaca alteration	Cloaca not quite closed, partially internal tissue shown	Cloaca prolapse	0.98	164
Keel bone damage	Noticeable deviation from the center line (0.5 - 1 cm), no callus or separation palpable	Clear palpable fracture (heavy deformation, mostly single-sided callus) or deviation from the center line > 1 cm	0.58	289
Toe injuries (both feet together)	Less than 3 small injuries	3 or more small injuries or one injury > 1 cm and/ or an amputated toe	0.89	164
Footpad dermatitis (worst foot)	(Mostly round) abscess or deviation without swelling or with swelling not visible from above	Swelling visible from above, mostly with footpad abscess	0.75	164
Laying impaired	Not clear if laying: mostly two fingers fit between the pubic bones, cloaca often smaller, belly smaller between pubis and keel bone	Non-laying: less than two fingers fit between the pubic bones, belly very small between pubic bones and keel bone	0.93	164
Ectoparasites found on the animal: poultry red mite, feather lice	yes	-	0.98	164

Information on management practices was collected through in-person interviews with the animal owner or a person responsible for the flock using structured and semi-structured questions (Table 3). Housing conditions in the mobile house, the covered veranda, if available, and free-range were recorded through inspection and assessment (Table 3). This included semi-quantitative evaluations of the air quality upon entering the house using a 3-point scale, and of litter condition at three locations in the scratching area (front, middle, rear) using a 4-point scale; the scores were averaged (Table 3). Space provision per hen, perch length, number of drinkers, nest space, and daylight inlet per floor area, were calculated based on the specifications provided by the mobile house manufacturers. Their construction drawings furthermore provided documentation of the design of the houses (arrangement of functional areas, presence of a covered veranda, structural and technical features). Mortality data were taken from the farm documentation.

**Table 3:** Management questionnaire and resource checksheet per mobile house.

		Answer/recording format
Questionnaire	Date of placement in the mobile house?	Date
	When and how long was the last time free-range access restricted due to HPAI?	Date, number of weeks
	To which extent is the mobile house integrated into the crop rotation?	1 = separate areas; 2 = part of crop rotation; 3 = dual-purpose use of the area, e.g. growing energy wood; 4 = other:
	Genetic origin of the flock?	Free answer
	Number of hens and number of cockerels	Free answer
	Did you molt your flock? When?	0 = no; 1 = yes, Date:
	Flock's use of the free-range area?	1 = whole free-range area; 2 = nearly whole area; 3 = half of the area; 4 = nearly half of the area; 5 = little more than the area near the house 6 = only area close to the house
	Planned relocation interval of the house?	Number of weeks during the growing season: Number of weeks outside the growing season:
	Time at current location?	Number of weeks:
	Decision criteria for moving the mobile house (single or multiple selection)	1 = regularly; 2 = availability of fresh pasture; 3 = condition of the area; 4 = trafficability of the area; 5 = fencing possible; 6 = other, namely:
	Keeping livestock guarding dog?	0 = no; 1 = yes
	Other livestock guarding animals? Which?	0 = no; 1 = yes, namely:
	Deterrence of predators by?	1 = light/reflection; 2 = sounds; 3 = flapping tape; 4 = scarecrow; 5 = other; 6 = none
Checksheet	Perches form and material (single or multiple selection)	1 = round and metal; 2 = rectangular or square, rounded and wooden; 3 = mix of round and metal with mushroom-shaped plastic
	Automatic feeding chain	0 = no; 1 = yes
	Additional trough to be filled by hand	0 = no; 1 = yes
	Additional trough not filled	0 = no; 1 = yes
	Automatic drinkers	0 = no; 1 = yes
	Water temperature (July to September)	°C, mean from the beginning, middle and end of the water supply
	Rollaway group nests	0 = no; 1 = yes
	Litter condition	1 = dry (material falls apart loosely or does not stick together); 2 = slightly moist: 3 = free-flowing but sticky; 4 = very moist: no longer free-flowing and can be formed into a lump; wet
	Floor plate	0 = no; 1 = yes
	Area under the mobile house accessible	0 = no; 1 = yes
	Ventilation of the house	1 = natural ventilation; 2 = mechanical ventilation; 3 = mix of natural and mechanical ventilation
	Air quality	1 = unimpaired or slightly impaired air quality (ammonia and/or dust not or barely perceptible); 2 = moderately impaired air quality (ammonia and/or dust perceptible); 3 = highly impaired air quality (acrid, burning)
	Location of the littered area	1 = inside the house; 2 = attached
	Covered veranda available	0 = no; 1 = yes
	Mobile house access without free-range entry	0 = no; 1 = yes
	Location of the free-range access	1 = access at ground level or below the house; 2 = access via ramps to elevated pop holes
	Free-range structured by artificial or natural elements	0 = no; 1 = yes

Fecal samples were examined for parasitic stages using a combination of direct smear, cytological straining (Diff-Quik™), flotation and sedimentation techniques. Native direct smears were prepared using sterile saline (NaCl 0.9%, B. Braun SE, Melsungen, Germany), and cytological evaluation was performed following Diff-Quik™ staining. For flotation, a saturated saline solution was used. Sediments were additionally examined for the presence of trematode and cestode stages. All samples were analyzed microscopically (Olympus BX46 Hamburg, Germany) at 200 × to 1,000 × magnification. Parasite burden was assessed semi-quantitatively based on the number of eggs or oocysts per field of 400 × magnification: low (1 egg or oocyst), moderate (2 - 5 eggs or oocysts), or high (> 5 eggs or oocysts).

In the altogether 36 flocks in stationary houses, animal-based welfare indicators (MTool©; Table 2) were repeatedly recorded (FR: n = 54 assessments, NR: n = 62 assessments) in the same way as described above by the person acting as silver standard and partly by further trained persons (employees of the project and the farms). Training included at least five joint assessments of 50 to 100 birds each on different farms and across different age groups, until employees and farmers were deemed proficient by the trainer. Additional assessments were conducted during the project at network meetings on various farms to allow participants to observe a wider range of variation in animal-based welfare indicators. Although formal reliability testing was not performed in this applied project, ensuring correct use of the scoring scheme was an important goal. In total, 15 training sessions involving multiple farmers were conducted over the course of the project.

The intended assessments at placement, peak of laying, and end of the laying period were not always achieved due to an HPAI outbreak. Most flocks were scored two (39%) or three times (25%), while 11% were scored once and 11% four times because of a longer laying period. In 8.3% of flocks molting was performed, and one of these flocks was reassessed one month after the completion of molting. In four farms (14%), in addition to the four surveys conducted by the project team, further surveys were carried out by trained farmers (1 to 12 additional assessments).

### Data processing and statistics

Welfare outcomes with a prevalence of ≥ 4% Score 1 or 2 findings (i.e., ≥ 2 affected hens per 50) were compared between mobile and stationary housing, the latter with or without free-range access (MH, FR, NR). Model assumptions were initially assessed visually using Q–Q plots of residuals. Due to deviations from normality and the presence of outliers, a robust linear mixed-effects model was applied (Koller, 2016). In these models, outliers exceeding a distance of k = 1.345 are not squared-weighted, allowing robust estimation of model parameters without discarding data and preserving the validity of the inference (Koller, 2016). Flock age (week of life) was included as an additional fixed effect in all models, while flock nested within house was included as a random effect. Mortality was not included in the statistical analysis due to insufficient availability of farm-provided records.

Robust mixed-effects models were also applied to compare the different mobile housing categories (WS, WM, WL, SB), regarding the welfare outcomes, including flock age as a fixed effect and flock nested within house as a random effect. An exception was the indicator “injuries back” for which a negative binomial model was applied to account for the right-skewed distribution.

Analyses were conducted using R statistical software (version 4.2.2) with the package *robustlmm*. Model diagnostics were further evaluated using simulated residuals from the *DHARMa* package (Hartig, 2024), which indicated no relevant deviations from model assumptions. Pairwise comparisons were performed using the Tukey post-hoc test. Results are presented as least squares means (**LS** means) with standard errors (**SE**).

## Results

### System description – technical specifications of the mobile houses (Resources)

Flock sizes on the farms were partly lower than the maximum numbers specified by the manufacturer due to efforts to improve bird welfare and in response to rising energy and feed costs or decreasing demand. The occasionally very high stocking densities (in SB) result from manufacturers including the covered veranda in the usable area. However, this is only permitted if the veranda is continuously accessible, whereas some farms restrict access to daytime only to reduce the risk of predation. As a result, some SB houses do not meet the legally mandated space allowance per animal. The varying actual stocking densities led to some overlap in flock sizes between mobile house categories (Table 1). An overview of the housing characteristics of the categories is provided in Table 4. Most mobile house types were equipped with automated chain feeders and egg collection belts, only some of the smaller houses in categories WS and SB had manually operated troughs and nests. In contrast to other house types, WS were partly equipped with littered nests (61.5%). To meet the legal trough space requirements, additional manually filled troughs were available in 63% of all mobile houses; however, they were found empty or even removed during 97% of visits. All other basic legal requirements, such as animals per drinker, nest space, perch length per animal, and daylight inlet per floor area, were met. Water was available through automatic drinkers in all mobile houses, and on all tiers in WS and WL (100%), and almost all SB (92%), whereas only 25% of WM provided water on all tiers. Predominantly round metal perches were used, whereas the house types in WS had rectangular, rounded wooden perches. A littered area was provided inside the house in all cases for WS, WM and WL, and in 58% for SB. The remainder had attached covered verandas that were littered. In WS, the littered area was located on the lower floor level of the house (on the level of the free-range) with a low headroom (68 to 90 cm) and 24/7 access. This was possible due to the house being hydraulically lifted above the wheels for relocation. In contrast, in WM and WL the wheels underneath the house area provided a shaded free-range area for the hens.

**Table 4:** Husbandry characteristics (resources) per mobile house category and in total.

Variables (dichotomous)	Unit	WS (13 houses, 24 flocks, 46 visits)	WM (12 houses, 19 flocks, 40 visits)	WL (11 houses, 22 flocks, 44 visits)	SB (12 houses, 21 flocks, 40 visits)	N (total)	Mobile housing total
Perches: round and metal	%	0.0%	100%	100%	91.7%	48	68.8%
Perches: rectangular or square, rounded and wooden	%	100%	0.0%	0.0%	0.0%	48	27.1%
Perches: mix of round and metal with mushroom-shaped plastic	%	0.0%	0.0%	0.0%	8.3%	48	4.1%
Automatic feeding chain	%	69.2%	100%	100%	91.7%	48	88.0%
Additional trough to be filled by hand	%	76.9%	66.7%	100%	8.2%	48	62.5%
Automatic drinkers	%	100%	100%	100%	100%	48	100%
Water at all tiers	%	100%	25.0%	100%	91.7%	48	73.5%
Automatic roll-away nests	%	38.5%	100%	100%	100%	48	83.3%
Floor plate	%	100%	100%	100%	25.0%	48	93.8%
Only natural ventilation	%	38.5%	0.0%	0.0%	75.0%	48	29.2%
Littered area inside the house	%	100%	100%	100%	58.0%	48	85.4%
Covered veranda available	%	100%	0.0%	63.6%	66.7%	48	58.3%
Stocking density	animals/m²	6.6 ± 1.3 (4.4 - 9.5)	7.7 ± 1.6 (5.0 - 9.0)	7.1 ± 1.4 (3.2 - 9.3)	8.5 ± 2.4 (5.2 - 13.9)	86	7.2 ± 1.8 (3.2 - 13.9)
Feeding space/animal	cm/nimal	10.5 ± 3.2 (3.4 - 14.8)	11.9 ± 3.0 (7.9 - 15.4)	9.8 ± 2.2 (7.0 - 18.0)	10.6 ± 2.2 (5.9 - 14.1)	170	10.7 ± 2.8 (3.4 - 18.0)
Animals/drinker	number	6.8 ± 1.7 (3.6 - 9.4)	7.9 ± 1.2 (5.7 - 9.1)	8.2 ± 1.4 (3.9 - 10.0)	8.0 ± 1.4 (6.0 - 10.0)	170	7.7 ± 1.5 (3.6 - 10.0)
Perch/animal	cm/animal	20 ± 4 (16.0 - 26.0)	19 ± 3 (16.0 - 24.0)	18 ± 4 (14.0 - 38.0)	18 ± 3 (15.0 - 28.0)	170	19 ± 3 (14.0 - 38.0)
Animals/nest space	animals/m²	72.6 ± 12.0 (59.1 - 95.3)	92.0 ± 11.4 (65.6 - 101.8)	93.7 ± 18.3 (46.7 - 119.3)	88.3 ± 15.5 (53.4 - 113.3)	170	86.3 ± 16.9 (46.7 - 119.3)
Daylight inlet space/floor area	%	32.7 ± 8.6 (23.2 - 44.1)	9.7 ± 3.5 (5.6 - 13.9)	5.3 ± 0.3 (5.1 - 5.9)	19.5 ± 1.4 (17.0 - 20.3)	142^1^	17.0 ± 12.7 (5.1 - 44.1)
Water temperature (July to September)	°C	20.0 ± 4.8 (11.0 - 27.5)	19.9 ± 3.4 (16.0 - 25.0)	20.0 ± 2.8 (16.0 - 27.0)	19.4 ± 3.5 (15.0 - 28.5)	29^2^	19.8 ± 3.8 (11.0 - 28.5)
Free-range access at ground level or below the house	%	100%	66.7%	36.4%	100%	48	77.1%

WS: wheel-based small (217 - 350 hens); WM: wheel-based medium (300 - 665 hens); WL: wheel-based large (587 - 2,059 hens); SB: skid-based (200 - 1,984 hens).

1 Light inlet area varies depending on the position of movable curtains in some SB housing units.

2 Drinking troughs are inaccessible in some houses within the WM and WL categories.

For manure removal in all commercial house types with aviaries electrically driven manure belts were provided. The house types with floor system either a manually operated manure belt (two WS house types) or a hydraulic scraper (one WS house type) underneath the perforated floor area. In the SB house types without floor plate and with manure pit, the manure had to be mechanically removed from the ground after the houses were moved to fresh pasture. In all mobile house types, manure removal from the littered area and litter provision had to be done manually. Ventilation was achieved by automatic negative pressure ventilation in WM and WL. In the categories WS and SB, there were also house types with natural ventilation, using automatically controlled flaps or curtains. In WS, the solid doors and walls of the littered floor area could be opened or removed, allowing additional air exchange through the metal-mesh panels and converting this area into a covered veranda. Consequently, a covered veranda could be provided temporarily in all WS, and also in 33% of SB, and was permanently available in 8% of WM, 46% of WL and 33% of SB.

Access to the free-range area was primarily at ground level or via pop holes beneath the house. In WM, however, 33% of free-range access occurred via ramps outside the house, whereas in WL, 64% of access occurred via the covered veranda, followed by raised pop holes and open ramps outside the house.

### System description – management of the mobile houses

An overview of the management practices in the different categories of mobile houses is provided in Table 5.

**Table 5:** Management practices on the 42 investigated farms and 48 houses per mobile house category and in total.

Variables (dichotomous)	Unit	WS (13 houses, 24 flocks, 46 visits)	WM (12 houses, 19 flocks, 40 visits)	WL (11 houses, 22 flocks, 44 visits)	SB (12 houses, 21 flocks, 40 visits)	N (total)	Mobile housing total
Pullets placed in winter (October to March)	%	77.3%	72.5%	50.0%	57.5%	86	64.7%
Additional trough not filled	%	89.5%	100%	100%	100%	109^1^	97.4%
Litter condition: dry	%	75.0%	70.0%	81.8%	87.5%	170	78.8%
Unimpaired or slightly impaired air quality	%	97.7%	95.0%	63.6%	87.5%	170	84.7%
Flock kept indoors due to HPAI	%	0.0%	10.5%	27.3%	19.0%	86	14.0%
Molted flocks	%	8.3%	10.5%	13.6%	14.3%	48	11.6%
Deterrence of predators	%	46.2%	36.4%	41.7%	16.7%	48	35.4%
Livestock guarding dog	%	0.0%	9.1%	25.0%	0.0%	48	8.3%
Livestock guarding with goat	%	23.1%	45.5%	9.1%	0.0%	48	18.8%
Livestock guarding with sheep	%	0.0%	18.2%	8.3%	8.3%	48	8.3%
Livestock guarding with cattle in summer	%	7.7%	9.1%	0.0%	0.0%	48	4.2%
Mobile house access without free-range entry	%	80.4%	20.0%	63.6%	72.2%	170	60.0%
Use of the whole free-range area	%	67.4%	75.0%	36.4%	65.0%	163^2^	63.2%
Mobile house integrated in crop rotation	%	50.0%	27.3%	27.3%	18.2%	42	33.3%
Decision to relocate the house based on:							
- regular interval	%	36.4%	70.0%	46.15%	33.3%	42	46.5%
- availability of fresh pasture	%	54.5%	60.0%	15.4%	44.4%	42	41.9%
- trafficability of the area	%	90.9%	90.0%	61.5%	88.9%	42	81.4%
- fencing possible	%	9.1%	20.0%	23.1%	11.1%	42	16.3%
Planned relocation frequency during growing season	Weeks	3.2 ± 1.9 (0.5 - 8.0)	5.0 ± 3.8 (2.0 - 13.0)	8.6 ± 5.2 (0.5 - 26.0)	8.3 ± 4.3 (1.0 - 15.0)	88	6.2 ± 4.6 (1.0 - 26.0)
Planned relocation frequency outside the growing season	Weeks	5.5 ± 4.3 (2.0 - 21.5)	7.6 ± 5.7 (2.0 - 16.0)	11.5 ± 7.0 (0.5 - 30.0)	13.3 ± 7.3 (5.0 - 26.0)	82	9.0 ± 6.8 (1.0 - 30.0)
Realized relocation frequency during growing season	Weeks	4.8 ± 4.3 (0.5 - 22.0)	5.8 ± 5.2 (1.0 - 22.0)	10.8 ± 7.0 (1.5 - 26.0)	10.9 ± 3.8 (4.0 - 16.0)	88	7.9 ± 5.9 (1.0 - 26.0)
Realized relocation frequency outside growing season	Weeks	7.7 ± 4.3 (2.0 - 17.0)	8.1 ± 4.8 (2.0 - 17.0)	12.8 ± 8.9 (1.0 - 34.0)	18.4 ± 19.6 (2.0 - 86.0)	82	11.9 ± 12.3 (1.0 - 86.0)

WS: wheel-based small (217 - 350 hens); WM: wheel-based medium (300 - 665 hens); WL: wheel-based large (587 - 2,059 hens); SB: skid-based (200 - 1,984 hens).

1 Not all mobile houses included additional troughs, which must be filled manually.

2 Some flocks were without free-range access until the visit due to HPAI.

In 65% of the cases, farmers introduced new pullets during winter (October to March).

Dry litter was observed in 70% (WM) to 88% (SB) of visits. Air quality was evaluated as unimpaired during most farm visits, with 95% and 98% of the visits in WS and WM and 64% and 88% in WL and SB. Water temperature in summer (July to September) did not differ between the mobile housing categories (Mean: 19.8°C; Min.: 11.0°C, Max.: 28.5°C).

In 63% of visits, the farmers subjectively rated the free-range use of the hens as “completely over the entire area”. The lowest percentage was reported for WL with 36%, in comparison to the other mobile house categories (65% to 75%). The shaded area under the mobile house (WM and WL) was inaccessible to the hens in 20% of visits. Farmers blocked access either due to mislaid eggs or because hens remained under the house in the evening. Most of the free-range areas were structured by artificial or natural elements (82%). The decision to relocate the mobile houses depended on the condition and accessibility of the area, the intended relocation interval, the availability of fresh pasture at the current location, and the feasibility of fencing. The mean relocation interval was lower during the vegetation period (8 weeks) than outside of it (12 weeks). Relocation was less frequent in WL (mean during vegetation: 10.8 weeks, and outside: 12.8 weeks) and SB (10.9 and 18.4 weeks) compared to WS (4.8 and 7.7 weeks) and WM (5.8 and 8.1 weeks). On some farms, the mobile houses were integrated into the crop-rotation system (33%). To protect the flock from predators, part of the farms used irritation materials like scarecrows or flapping objects (35%) or livestock guarding animals (dogs, goats, sheep or cattle; 40%).

With regard to biosecurity, in 60% of the visits, the mobile house was placed directly at the fence, which permitted access to the house without crossing the free-range area. Three WS farms, where an anteroom was not available as a sanitation area, implemented their own solutions for storing barn shoes in lockable garbage bins or boxes placed in front of the house. In total, 14% of the flocks had been kept indoors for several weeks or months due to HPAI restrictions.

### Welfare outcomes

Mortality data for up to at least week 62 of life were available from 20 mobile housed flocks, with causes of losses only recorded in 9 flocks. The mean documented mortality rate was 10.0% (1.6 - 22.6%), and predators were the most frequently cited cause (44.9%).

Eggs or oocysts from endoparasites were found in 45.5% of the fecal samples with a predominantly low burden (Table 6), and mostly from *Heterakis/Ascaridia* species (91.9%). Mites were found on a mean of 1%, and feather lice on 2% of the assessed hens (Table 6).

**Table 6:** Results per mobile house category of endoparasite analysis (pooled fecal samples from 167 visits, three farms declined analysis during three visits) and prevalences (%) of red poultry mites or feather lice on the hens (n = 50); taken from 86 flocks during 170 farm visits.

Parasite infestation	WS (45 visits)	WM (40 visits)	WL (43 visits)	SB (39 visits)	Mobile housing total (167 visits)
*Endoparasites*					
Negative	52.3%	63.4%	41.9%	61.5%	54.5%
Low grade (1 egg or oocyst^1^)	43.2%	36.6%	58.1%	38.5%	44.3%
Medium grade (2 to 5 eggs or oocysts^1^)	2.3%	0.0%	0.0%	0.0%	0.6%
High grade (> 5 eggs or oocysts^1^)	2.3%	0.0%	0.0%	0.0%	0.6%
*Ectoparasites*					
Red poultry mite on the hens	0.2 ± 0.9 (0 - 6)	2.7 ± 11.5 (0 - 60)	1.4 ± 5.7 (0 - 34)	0.6 ± 2.7 (0 - 14)	1.2 ± 6.5 (0 - 60)
Feather lice on the hens	2.6 ± 12.2 (0 - 72)	1.3 ± 8.1 (0 - 52)	3.7 ± 14.5 (0 - 78)	0.0 ± 0.0 (0 - 0)	2.0 ± 10.6 (0 - 78)

WS: wheel-based small (217 - 350 hens); WM: wheel-based medium (300 - 665 hens); WL: wheel-based large (587 - 2,059 hens); SB: skid-based (200 - 1,984 hens).

1 Per 400 x magnification.

Mean prevalences of eye alterations, respiratory infections, and cloaca prolapse were low across all housing categories ranging from 0 to 3.6% (sum of Score 1 and 2, Supplementary Table 1). Within the mobile housing categories, pale combs occurred more frequently in WL, compared to WS and WM, and feces soiling at the back more frequently in WS compared to WM. Also, prevalences of footpad dermatitis were significantly higher in WS compared to all other categories. Otherwise, there were no significant differences between the mobile house categories (Table 7).

**Table 7:** Comparison of prevalences of different welfare problems (Score 1 and 2, assessed using MTool©, Table 2) between four categories of mobile housing: least square means ± standard errors, different superscripts indicate significant differences (p < 0.05).

Welfare problems 1	η² 2	WS	WM	WL	SB
Pale comb	0.068	4.06 ± 1.23 ^a^	3.27 ± 1.31 ^a^	10.75 ± 1.29 ^b^	6.32 ± 1.32 ^a,b^
Comb & wattle injuries	0.010	56.0 ± 8.47	39.2 ± 9.17	60.9 ± 9.40	52.1 ± 9.20
Plumage damage neck	0.049	18.1 ± 2.26	11.8 ± 2.42	11.1 ± 2.31	13.2 ± 2.42
Plumage damage back	0.019	10.2 ± 3.55	12.2 ± 3.92	19.6 ± 3.69	13.9 ± 3.82
Injuries back	0.029	4.90 ± 3.03	2.22 ± 1.51	10.12 ± 6.40	0.92 ± 0.65
Feces soiling back	0.079	16.71 ± 2.88 ^a^	3.38 ± 3.03 ^b^	7.26 ± 3.10 ^a,b^	10.15 ± 3.02 ^a,b^
Plumage damage belly	0.021	3.83 ± 0.79	3.47 ± 0.85	4.53 ± 0.81	5.53 ± 0.85
Injuries belly	0.006	2.59 ± 0.50	1.88 ± 0.54	2.97 ± 0.51	2.46 ± 0.54
Feces soiling belly	0.005	18.9 ± 3.43	15.0 ± 3.81	16.0 ± 3.57	19.4 ± 3.69
Keel bone damage	0.019	48.2 ± 3.70	37.7 ± 3.91	46.3 ± 3.92	49.2 ± 3.93
Footpad dermatitis	0.174	22.0 ± 2.43 ^a^	7.19 ± 2.58 ^b^	10.18 ± 2.56 ^b^	8.28 ± 2.59 ^b^

WS: wheel-based small (217 - 350 hens); WM: wheel-based medium (300 - 665 hens); WL: wheel-based large (587 - 2,059 hens); SB: skid-based (200 - 1,984 hens).

1 Indicators with prevalence of Score 1 or 2 ≥ 4%.

2 Effect size as Eta² = η².

Significantly lower prevalences of plumage damage at the back and neck were observed in mobile systems (MH) compared to both stationary systems (FR and NR). The same applied to plumage damage at the belly and beak alterations, both with even higher prevalences in stationary houses without free-range access (NR) than in stationary houses with free-range access (FR). Injuries at the back and belly were only significantly increased in NR which also was the case for toe injuries and crop alterations. In contrast, prevalences of comb and wattle injuries were lower in NR than in MH. Prevalences of pale or bluish combs were significantly increased in FR. No differences between systems were found for soiled plumage, belly inflammation, keel bone damage and footpad dermatitis (Table 8). Overall, welfare outcomes showed considerable variation between flocks. For all animal-based indicators, Score 1 or 2 prevalences of 0% were found, but also higher prevalences, occasionally up to 100% in all housing categories (Supplementary Table 1).

**Table 8:** Comparison of prevalence of welfare problems (Score 1 and 2, assessed using MTool©, Table 2) between mobile and stationary systems: least square means ± standard errors, different superscripts indicate significant differences (p < 0.05).

Welfare problems 1	η² 2	MH	FR	NR
Beak alterations	0.829	0.84 ± 0.70 ^a^	14.92 ± 1.24 ^b^	48.64 ± 1.15 ^c^
Pale comb	0.086	6.62 ± 1.05 ^a^	17.23 ± 1.84 ^b^	8.86 ± 1.72 ^a^
Bluish comb	0.032	0.45 ± 0.10 ^a^	1.35 ± 0.28 ^b^	0.29 ± 0.31 ^a^
Comb & wattle injuries	0.029	51.30 ± 4.67 ^a^	26.90 ± 14.00 ^a,b^	12.20 ± 15.70 ^b^
Crop alterations	0.450	0.00014 ± 0.000088 ^a^	0.00091 ± 0.00015 ^b^	0.0026^±^ 0.00014 ^c^
Plumage damage neck	0.120	15.90 ± 1.89 ^a^	27.30 ± 3.57 ^b^	37.80 ± 3.56 ^b^
Plumage damage back	0.068	18.10 ± 3.36 ^a^	38.60 ± 7.02 ^b^	47.10 ± 7.19 ^b^
Injuries back	0.075	0.78 ± 0.21 ^a^	1.24 ± 0.41 ^a^	2.92 ± 0.41 ^b^
Feces soiling back	0.016	10.42 ± 1.99	8.77 ± 5.73	24.32 ± 6.37
Plumage damage belly	0.359	6.36 ± 1.42 ^a^	14.88 ± 3.13 ^b^	50.77 ± 3.34 ^c^
Injuries belly	0.159	2.80 ± 0.50 ^a^	4.86 ± 0.87 ^a^	9.43 ± 0.81 ^b^
Feces soiling belly	0.004	16.80 ± 1.96	15.20 ± 5.66	10.00 ± 6.31
Inflammation belly	0.017	0.68 ± 0.12	0.75 ± 0.21	1.16 ± 0.19
Keel bone damage	0.016	45.80 ± 2.18	32.3 ± 5.98	42.8 ± 6.63
Footpad dermatitis	0.001	11.60 ± 1.26	10.2 ± 3.18	13.3 ± 3.49
Toe injuries	0.771	0.48 ± 0.17 ^a^	0.88 ± 0.29 ^a^	10.32 ± 0.27 ^b^

MH: Mobile housing systems; FR: stationary houses with free-range access; NR: stationary houses without free-range access.

1 Indicators with prevalence of Score 1 or 2 ≥ 4%.

2 Effect size as Eta² = η².

## Discussion

To our knowledge, this is the first observational study to investigate different categories of mobile houses in terms of potential hazards and welfare outcomes in farming practice, including a comparison with stationary systems.

By using the MTool© protocol, developed for early detection of animal welfare issues, the number of indicators considered was larger than in many other studies. The potential welfare problems addressed include key issues identified in recent studies (reviewed by EFSA AHAW Panel et al., 2023). Some other welfare indicators were expected to be relevant only in individual cases. Indeed, eye alterations, respiratory infections, cloacal prolapse and impaired laying occurred at low mean prevalences (< 4%) across all housing systems, whereas bluish combs, crop alterations, belly inflammation, and toe injuries reached mean prevalences of up to 9.5% in the stationary systems. The occurrence of higher prevalences (up to 100%) in some of these indicators on individual farms in all housing systems (MH, FR, NR), however, support their inclusion in an animal welfare assessment. Although the distinction between lower and higher prevalence is necessarily arbitrary, we used 4% as a threshold, as this corresponds to two birds in the sample of 50.

### Comparison between mobile house categories - housing and management aspects

In some SB houses, attached covered verandas lead to stocking densities exceeding the legal maximum, as the verandas are closed at night. To ensure continuous accessibility and thus compliance with legal requirements, the covered verandas would need to be predator-proof. Air quality was more often rated as moderately or highly impaired in WL (36.4%) than in the other mobile housing categories (WS: 2.3%, WM: 5.0%, SB: 12.5%). Although the subjective one-time assessment is certainly questionable in terms of repeatability, and quantitative long-term measurements would have been preferable, the assessment in the rough categories used provides at least first indications that sufficient air exchange in larger flocks, even with mechanical ventilation, may be challenging to achieve. Apparently, in the SB systems with also larger flocks, but typically natural ventilation with large side curtains and high insulated ceilings (75.0%), it may be easier to control air quality due to the greater air volume per bird. Although the littered area in SB was directly on the ground, the proportion of dry litter was also highest, which may have interacted with the better air quality and was also due to measures to prevent rainwater from entering such as trenches or sandbags.

Adequate air exchange is particularly crucial during high summer temperatures. Heat stress is a significant issue in poultry farming (Teeter and Belay, 1996; Scanes and Sturkie, 2014; Goel, 2021), with mobile housing being more susceptible than stationary housing, due to lower air volume, less insulation, and restricted technical options of mechanical ventilation (van der Linde and Pieper, 2018). Providing cool drinking water (13 - 15°C) during hot weather can lower body temperature, increase feed intake, and improve eggshell quality compared to ambient-temperature water (Teeter and Belay, 1996; Glatz, 2001). In summer 2022, mean water temperature in the studied mobile houses (20°C) was below the ambient temperature, but above recommended levels, indicating a need for strategies to improve cooling. To reduce heat stress in young hens and facilitate adaptation to an increasing photoperiod, most flocks (64.7%) were placed during winter.

### Comparison between mobile housing categories - welfare outcomes

Very few differences in welfare outcomes were detected between mobile housing categories. Pale combs were more prevalent in WL with larger flocks. Following indications from stationary housing (reviewed by Knierim, 2006; Whay et al., 2007, Gebhardt-Henrich et al., 2014; Gilani et al., 2014; Bestmann et al., 2019), birds in larger flocks use the free-range area less than in smaller flocks. Some studies link pale combs to reduced free-range access (Whay et al., 2007; Bestman and Wagenaar, 2014), and Larsen et al. (2018) found that hens with pale combs were more likely to never go outside than hens with red combs. This aligns with farmers’ reports that WL flocks used the entire free-range area least frequently. Elevated pop holes in WL (72.7%) may impair free-range access, as hens could perceive ramps as less secure than ground-level exits. On the other hand, results may be biased, because it is harder to assess comb color under low light intensity and with less natural light. House size and covered verandas in 54.5% of WL reduced indoor daylight.

Feces soiling on the back occurred most in WS and least in WM, the latter being equipped with aviaries with manure belts, while perches in WS were arranged vertically, which apparently poses a higher soiling risk. However, most of the soiling fell into Score 1, which describes plumage discoloration or minimal feces adhesion, and might therefore be of lower welfare concern.

In WS, a higher proportion of birds with footpad dermatitis was observed compared to the other categories. One notable difference was the use of rectangular or square wooden perches instead of round metal ones. It is well established that hens prefer the rectangular perch shape (Chen et al., 2014). Less foot damage compared to circular perches has been reported from enriched cages (Duncan et al., 1992), although the pressure on the hens’ footpads during standing and sitting does not differ between round and square perches (Pickel et al., 2011). Moreover, wooden perches pose a risk of injury due to splinters and can be associated with increased perch soiling (Wang et al., 1998), increasing the risk of footpad damage. In addition, moist litter, identified in 25% of WS farm visits, may have contributed to higher prevalences, being a major risk factor for impaired footpad health (Wang et al., 1998). Difficult accessibility of the littered area for the farmers may reduce the frequency of removal of moist litter or topping up of dry litter in this housing category. However, in WM even in 30% of farm visits litter was assessed as moist, but the prevalence of footpad dermatitis was significantly lower than in WS. According to Volkmann et al. (2024), the direct impact of moist litter on laying hen footpad health remains inconclusive. Further research on footpad health in laying hens should be undertaken.

Against expectations that the provision of rectangular or square wooden perches in WS versus round metal perches in the other categories could have a mitigating effect on keel bone damage (Pickel et al., 2011), no differences in prevalence between the housing categories were detected.

In mammals, pasture rotation can limit endoparasite infestation, but relevant data on effective range rest periods are lacking for poultry systems (reviewed by Bonnefous et al., 2022). In a small two-year study, Wongrak et al. (2014) observed egg-positive samples increasing from almost none in the first year to on average 47% in the second, despite each mobile house location being used no more than twice a year. Environmental persistence of parasite eggs may further constrain rotation effectiveness: *Ascaridia galli* and *Heterakis gallinarium* eggs can remain viable for several months, with a small proportion persisting for up to two years (Thapa et al., 2017). Infection likely occurs outdoors (Höglund and Jansson, 2011), though contaminated feces may also be transferred to new areas within or via the mobile house. In the present study, the generally low endoparasite burden prevented comparisons between mobile housing categories: 52% of samples were negative and 44% showed low burdens, mainly *Heterakis/Ascaridia*, which is lower than reported for stationary free-range systems with hens of comparable or older age (Kaufmann et al., 2011; Thapa et al., 2015; Bestman et al., 2023). Farms had operated mobile housing for on average 4.6 years, indicating that the low infection rates cannot be explained by predominantly newly established ranges. Variation may arise from diagnostic methods (Ballweber et al., 2014; Zloch et al., 2021), and increasing prevalences with bird age (Zloch et al., 2021). A certain parasite burden is unavoidable in free-range systems, but the levels observed here are unlikely to pose serious welfare concerns.

Relocation intervals in WS and WM averaged 5 weeks, which was about half the length of those in WL and SB. Over all categories, mobile houses were on average relocated every 8 weeks during the growing season and every 12 weeks outside it. The interval was often longer than planned (2 - 3 weeks) due to no trafficability of the range (81%), no availability of fresh pasture (42%) and impaired possibility of fencing (16%). Relocating is avoided in wet conditions to prevent sward damage, while frost or prolonged summer drought can hinder fencing. To avoid long-term destruction of pasture in well-used free-range areas, bi-weekly relocation is recommended in the vegetation period and every six weeks outside the vegetation period (Fürmetz et al., 2005a). This moreover prevents nutrient leaching (Fürmetz et al., 2005b; Heß and Deerberg, 2017). Only 33% of farms integrated mobile houses into crop rotation, indicating untapped potential.

Red bird mites typically hide near hens' resting areas during the day; traps are commonly used to assess infestation (Murillo et al., 2020). Due to the long visit intervals, no traps were used in this study. Visible mites on hens during daytime indicate high infestation (Keppler and Knierim, 2020a), as observed in individual mobile-housed flocks. The complex environment of mobile houses, could promote infestations (reviewed by Sigognault Flochlay et al., 2017). Mitigation potential related to house design as well as cleaning and disinfection requires further investigation.

### Comparison between mobile and stationary housing systems – welfare outcomes

Feather damage on the neck, back, and belly was significantly lower in MH compared to FR and NR, with most cases mild (Score 1) and below levels reported from other studies for stationary systems. For example, Campe et al. (2018) found more severe feather damage (comparable to Score 2) in beak-trimmed stationary flocks without free-range access (16.8% neck, 22.9% back, 25.6% belly) than in MH (3.0%, 5.9%, 2.5%), while FR (16.1%, 30.3%, 18.8%), and NR (13.2%, 24.9%, 23.6%) were at a similar level in this study. Schreiter et al. (2020), reported that 59.0% of cage-free birds with intact beaks had mild feather damage (Score 1) when combining scores on belly and back by considering the worst score. Heerkens et al. (2015) reported higher prevalences of 70.1% feather damage at the back and 56.4% at the belly (Scores 1 and 2) in predominantly beak-trimmed flocks with and without free-range access. Schwarzer et al. (2022) observed an average of 30.7% affected non-beak-trimmed hens in stationary flocks with and without free-range access, with prevalence increasing with age to 57.8% (Score 1 and 2). This trend was also observed in other studies (Schreiter et al., 2020; van Staaveren et al., 2021) and aligns with our findings. If feather pecking starts, it can quickly spread in a flock (Bestman et al., 2017; Campe et al., 2018), as reflected by the large range of prevalences across all three housing systems in this study. Feather pecking is a multifactorial behavioral problem. High free-range use is an important mitigation factor (reviewed by Knierim, 2006; Rodenburg et al., 2013; Jung and Knierim, 2018), and the availability of forage attracts hens to the free range (Nagle and Glatz, 2012). Although many factors may have contributed to the differences between mobile and stationary systems, more attractive free-range areas and smaller flock sizes, which promote better free-range use, were likely relevant. This also applies to the lower belly plumage damage in stationary flocks with free-range access compared to NR. However, for flocks accustomed to free-range access, mandatory housing during HPAI remains a major hazard. Especially in mobile systems, creative solutions such as access to an open barn or an attached covered veranda may be required.

Pecking injuries on the back and belly were in general less frequent, consistent with other studies (Nicol et al., 2006; Lambton et al., 2015). Here only NR had significantly higher prevalences than both MH and FR, pointing again at the importance of free-range access. In general, prevalences were below values reported in the literature from 13.0% to 29.9% (Heerkens et al., 2015; Bestman et al., 2017; Schreiter et al., 2020; Jung et al., 2024). Similar mitigating factors to those for feather damage may apply to cannibalism (Pötzsch et al., 2001; Knierim, 2006; Bestman et al., 2017), though fewer evidence is available.

The significantly higher prevalence of comb and wattle injuries in MH (Score 1 and 2: 50.9%) compared to FR and NR is challenging to interpret, as such injuries are rarely studied. Scores 1 and 2 are comparable to the Welfare Quality® Score 2 used in other studies, although the latter excludes wattle injuries and may underestimate prevalence. Most injuries likely result from agonistic pecking (Eklund and Jensen, 2011; Welfare Quality, 2019) linked to resource competition (Weimer et al., 2019; Bari et al., 2020). Reported prevalences range from 0% and 1% (Nenadović et al., 2022; Göransson et al., 2023) to 40% (Blatchford et al., 2016; Nenadović et al., 2022). As injuries are typically small, differences in lighting during assessment may affect detection. Greater natural light in MH likely improved detection. Similar prevalences across all mobile housing categories suggests no flock size effect, as also noted by Nenadović et al. (2022). Resource competition may have been exacerbated by unfilled supplementary troughs. Although 62.5% of houses provided additional manually refillable troughs to meet legal requirements (TierSchNutztV, 2006), 97.4% remained empty. This indicates a welfare risk associated with manual feeding, favoring automated systems. Further research on drivers of agonistic pecking is warranted.

The significantly lower occurrence of beak alterations (e.g., overlong or broken) in MH compared to FR, and FR compared to NR may be associated with increasing beak use for foraging behavior in the free-range systems, although other influences cannot be excluded. However, beak condition might therefore be an interesting indicator of use of foraging enrichment or the free-range by the hens and should be further investigated.

Pale or bluish combs were significantly more prevalent in FR than in NR and MH, suggesting that in FR possible effects of health factors (Bestman and Wagenaar, 2014; Bakar et al., 2023) were more relevant than the effect of free-range access. However, also hybrid type determines comb color (Bestmann and Wagenaar, 2014), which could have affected the results, with mainly white hybrids (72.6%) in NR. Moreover, as already mentioned, assessments conducted in confined indoor versus naturally lighted environments may be of limited comparability.

Keel bone damage occurred at prevalences comparable to previous studies (Blatchford et al., 2016; Jung et al., 2020; Rufener and Makagon, 2020, Jung et al., 2024) with no statistically significant differences between systems. Consistent with earlier findings, prevalence increased with age (Donaldson et al., 2012; Petrik et al., 2015; Hinrichsen et al., 2016; Jung et al., 2019; Eusemann et al., 2020). Potential benefits of high free-range use areas, such as improved bone strength (reviewed by Knierim, 2006; Sandilands et al., 2009; Wilkins et al., 2011), may be offset by a higher risk of accidents associated with elevated structures (Stratmann et al., 2015; Riber and Hinrichsen, 2016; Jung et al., 2019) in the long, narrow mobile houses. Optimizing mobile house design to reduce accident risk should therefore be a priority for mobile house manufacturers.

Levels of plumage soiling with feces and footpad dermatitis did not significantly differ between mobile and stationary systems due to large variation within systems, suggesting an important role of management factors.

### Limitation of the study

In addition to the limitations discussed above, it should be noted that all farmers participated voluntarily. MH, FR and NR farms were part of two projects in which the husbandry and management conditions were continuously evaluated and improved. Therefore, these farms may have been more engaged concerning animal welfare than average, although welfare outcomes still showed a wide range. Moreover, the comparison between mobile and stationary houses is limited due to the data originating from two separate studies that were carried out in different years. Despite different data collection time points in the two studies, the mean age in weeks of life was very similar. Furthermore, results from the stationary flocks align well with those of other published studies. Training and inter-assessor agreement testing concerning the mobile housing data were conducted with the same person who collected part of the stationary housing data. For the remaining data recording, no formal inter-assessor agreement testing was performed; however, the project employees and farmers were thoroughly trained - both theoretically and practically - by the same person.

It was not feasible to select farms based on the genetic origin; instead, the aim was to reflect the typical diversity of German mobile laying hen systems. Due to the frequent use of alternative marketing channels, a higher prevalence of rare breeds or crosses from an organic breeding company were expected. However, brown hybrids predominated. The heterogeneity of genetic backgrounds across farms and flocks precluded their systematic consideration in the analyses and genetic influences on the results cannot be excluded. This is particularly relevant when comparing housing systems: in NR, the proportion of white hybrids was high (72%), whereas none were present in FR and only 8% in MH (exclusively in mixed flocks). Feather pecking shows genetic components (Kjaer and Sørensen, 1997; Kjaer et al., 2001; Keppler, 2008; Iffland et al., 2020). Some studies reported higher prevalences in brown than white genotypes (Kjaer and Sørensen, 1997; Kjaer, 2000; de Haas et al., 2014), others the opposite (de Haas et al., 2013; Habig and Distl, 2014; Bestman et al., 2017), or no differences (Campe et al., 2018; Decina et al., 2019). This may be explained by effects of differing environmental conditions (Cronin and Glatz, 2020; Mens et al., 2023). Furthermore, it should be considered that genetic differences may also occur within white or brown hybrids due to ongoing breeding (Nicol, 2018; Coton et al., 2019). Toe pecking was exclusively observed in white hybrids by Gebhardt-Henrich et al. (2023), which is consistent with our findings of higher prevalences in NR. However, risk factors of toe pecking are barely investigated (Mens et al., 2023).

Molting became more relevant during the data collection period for mobile housing due to rising energy and feed prices; 10 of 86 mobile-housed flocks underwent molting, whereas only one of the 18 FR and two of the 18 NR flocks were molted. The welfare effects of molting are difficult to predict and largely depend on the molting method applied (McCowan et al., 2006). The flocks were visited at least one month after molting and numerically showed no conspicuous differences in the prevalences of feather damage or injuries at the back and belly compared to other MH flocks.

More comprehensive data on mortality, its causes, and laying performance would have been desirable. Despite a legal requirement of documentation, however, we received only very limited access to records. The reported average mortality of 10% should therefore be interpreted with caution. Previous studies suggest that predation accounts for a substantial portion of losses in free-range systems (Hegelund et al., 2006; Elson, 2015; Singh et al., 2017), which was confirmed by the participating farmers. Although various predator deterrence measures were reported, further research is needed. While laying performance records were available for 55 of 86 flocks, the lack of mortality data prevented reliable calculation of laying performance measures.

When interpreting the results of this observational study, it should be considered that they are strongly influenced by the individual housing conditions and management practices as well as the genetic background and age of the birds. This limits the external validity of conclusions on welfare potentials of different housing categories or systems, which strictly only apply to the flocks investigated. However, as our sample of commercial farms reflects to some degree the large between-farm variation within the same housing category, it nevertheless provides first indications of opportunities and challenges of mobile housing systems in farming practice.

## Conclusion

Compared to stationary systems, mobile housing showed a potential to mitigate feather pecking and cannibalism, while individual problems can occur in any system. This may underline the importance of high free-range use as mitigating factor. The higher prevalence of comb and wattle injuries recorded in mobile houses raises questions about the underlying factors influencing agonistic pecking. Other welfare problems such as footpad or keel bone damage, appear to be unaffected by mobile versus stationary system.

Relatively few differences in welfare outcomes among the four mobile housing categories suggest that appropriate management is more important than the housing category itself. The causes of specific differences should be investigated further, particularly for pale combs (most common in WL), footpad dermatitis (most common in WS), and feces soiling at the back (less common in WM).

The identified welfare challenges include effective predator protection, provision of appropriate enrichment during periods of restricted free-range access due to HPAI, effective climate control in large wheel-based mobile houses, providing sufficient indoor feeding space per hen, even if this requires increased manual labor, and relocating the mobile units at optimal intervals.

## CRediT authorship contribution statement

**K. Dorkewitz:** Writing – review & editing, Writing – original draft, Validation, Methodology, Investigation, Formal analysis, Data curation, Conceptualization. **C. Keppler:** Writing – review & editing, Validation, Methodology, Investigation, Data curation. **D. Gieseke:** Writing – review & editing, Validation, Project administration. **S. Horbach:** Writing – review & editing, Investigation, Data curation. **U. Knierim:** Writing – review & editing, Supervision, Project administration, Methodology, Funding acquisition, Conceptualization.

## Disclosures

The authors declare that they have no known competing financial interests or personal relationships that could have appeared to influence the work reported in this paper.

## Appendix Supplementary materials

Image, application 1

## Footnotes

Supplementary material associated with this article can be found, in the online version, at doi:10.1016/j.psj.2026.106992.
