# Supplementary material for: Opportunities and challenges of modern mobile housing systems for laying hen welfare - an observational study
Source: Poult Sci. 2026 Apr 22;105(8):106992. doi: 10.1016/j.psj.2026.106992 (PMC13196515; doi:10.1016/j.psj.2026.106992)
Supplement: Supplementary file 1 [file mmc1.docx]

Supplemental Table 1: Prevalences of different welfare problems (assessed using MTool©) in mobile housing (MH), stationary housing with (FR) and without free-range access (NR) (mean ± standard deviation, min–max). For score definitions, see Table 2; measures with a prevalence ≥ 4.0% in one or more housing systems are highlighted in bold.

|  | MH  n = 170 farm visits | | FR  n = 54 farm visits | | NR  n = 62 farm visits | |
| --- | --- | --- | --- | --- | --- | --- |
|  | Score 1 + 2 | Score 2 | Score 1 + 2 | Score 2 | Score 1 + 2 | Score 2 |
| **Beak alteration** | **1.0 ± 2.8**  (0 - 24) | **0.6 ± 2.1**  (0 - 22) | **26.9 ± 28.9** (0 - 86) | **2.8 ± 3.3**  (0 - 12) | **47.4 ± 26.5** (0 - 100) | **6.9 ± 11.2**  (0 - 50) |
| Eye alterations | 0.2 ± 0.8  (0 - 6) | 0.1 ± 0.5  (0 - 4) | 0.5 ± 2.0  (0 - 14) | 0.0 ± 0.0  (0 – 0) | 1.6 ± 7.6  (0 - 56) | 0.5 ± 3.5  (0 - 28) |
| [Respiratory infection](https://www.linguee.de/englisch-deutsch/uebersetzung/respiratory+infection.html) | 0.0 ± 0.0  (0 - 0) | 0.0 ± 0.0  (0 - 0) | 0.0 ± 0.3  (0 - 2) | 0.0 ± 0.0  (0 - 0) | 0.0 ± 0.0  (0 - 0) | 0.0 ± 0.0  (0 - 0) |
| **Pale comb** | **8.5 ± 12.8** (0 - 58) | **0.6 ± 1.8**  (0 - 12) | **27.0 ± 30.6** (0 - 96) | **3.2 ± 9.4**  (0 - 62) | **16.1 ± 26.3** (0 - 100) | **2.4 ± 13.2** (0 - 100) |
| **Bluish comb** | **1.3 ± 3.0**  (0 - 18) | **0.1 ± 0.5**  (0 - 4) | **5.9 ± 10.1** (0 - 34) | **0.8 ± 4.2**  (0 - 12) | **5.0 ±19.6** (0 - 100) | **3.9 ± 17.4** (0 - 100) |
| **Comb & wattle injuries** | **50.9 ± 32.8** (0 - 100) | **18.1 ± 15.8** (0 - 74) | **33.0 ± 35.4** (0 - 100) | **10.9 ± 19.6** (0 - 76) | **9.9 ± 21.2** (0 - 96) | **2.5 ± 12.1** (0 - 88) |
| **Crop alterations** | **0.0 ± 0.3**  (0 - 2) | **0.0 ± 0.0**  (0 - 0) | **0.5 ± 2.0**  (0 - 14) | **0.1 ± 0.5**  (0 - 2) | **16.9 ±35.2** (0 - 100) | **9.5 ±22.2** (0 - 84) |
| **Plumage damage neck** | **17.3 ±24.8** (0 - 100) | **3.0 ± 9.5**  (0 - 84) | **30.6 ± 33.8** (0 - 100) | **11.9 ± 20.9** (0 - 92) | **48.3 ± 40.0** (0 - 100) | **22.2 ± 27.5** (0 - 92) |
| **Plumage damage back** | **18.3 ± 27.7** (0 - 98) | **5.9 ± 16.2** (0 - 82) | **39.8 ± 40.6** (0 - 100) | **24.0 ± 33.7** (0 - 98) | **52.1 ± 42.0** (0 - 100) | **28.5 ± 34.0** (0 - 100) |
| **Injuries back** | **2.4 ± 7.8**  (0 - 56) | **0.6 ± 2.9**  (0 - 30) | **5.7 ± 15.1** (0 - 88) | **2.3 ± 9.1**  (0 - 62) | **14.6 ±21.6** (0 - 85) | **2.9 ± 8.7**  (0 - 52) |
| **Feces soiling back** | **12.5 ± 17.7** (0 - 80) | **1.2 ± 2.8**  (0 - 14) | **11.4 ± 16.2** (0 - 68) | **2.3 ± 5.1**  (0 - 22) | **36.4 ± 36.1** (0 - 100) | **11.1 ± 19.2** (0 - 86) |
| **Plumage damage belly** | **9.0 ±18.9** (0 - 98) | **2.5 ± 8.8**  (0 - 84) | **22.7 ± 33.8** (0 - 100) | **13.7 ± 28.9** (0 - 92) | **52.9 ± 40.0** (0 - 100) | **31.0 ± 35.6** (0 - 100) |
| **Injuries belly** | **4.4 ± 9.9**  (0 - 68) | **1.0 ± 3.7**  (0 - 30) | **9.1 ± 15.0** (0 - 70) | **2.1 ± 5.9**  (0 - 32) | **20.9 ± 26.5** (0 - 100) | **6.3 ± 12.3** (0 - 52) |
| **Feces soiling belly** | **19.1 ± 19.1** (0 - 92) | **2.7 ± 8.0**  (0 - 72) | **14.6 ±15.7** (0 - 64) | **1.1 ± 2.7**  (0 - 14) | **9.9 ± 14.7** (0 - 64) | **1.9 ± 4.8**  (0 - 24) |
| **Inflammation belly** | **1.7 ± 5.2**  (0 - 40) | **1.0 ± 4.5**  (0 - 36) | **3.3 ± 9.3**  (0 - 46) | **0.4 ± 1.3**  (0 - 8) | **6.0 ±13.7** (0 - 76) | **0.4 ± 1.7**  (0 - 10) |
| Cloaca alteration | 0.1 ± 0.4  (0 - 4) | 0.0 ± 0.3  (0 - 2) | 1.6 ±4.5  (0 - 22) | 0.5 ±2.7  (0 - 20) | 3.6 ± 11.8 (0 - 74) | 0.6 ± 2.6  (0 - 16) |
| **Keel-bone damage** | **45.2 ± 24.1** (0 - 94) | **31.6 ± 20.7** (0 - 84) | **34.4 ±21.4** (4 - 86) | **16.1 ± 15.7** (0 - 78) | **36.4 ± 22.2** (4 - 88) | **16.2 ± 19.5** (0 - 76) |
| **Toe injuries** | **1.0 ± 3.9**  (0 - 39) | **0.2 ± 1.1**  (0 - 10) | **1.1 ± 2.0** (0-8) | **0.2 ± 0.7**  (0 - 4) | **26.7 ± 18.3** (0-66) | **3.1 ± 7.6**  (0 - 38) |
| **Footpad dermatitis** | **13.8 ± 16.5** (0 - 80) | **0.7 ± 2.2**  (0 - 22) | **13.9 ± 15.4** (0-84) | **1.2 ± 4.6**  (0 - 30) | **14.8 ± 14.3** (0 - 52) | **1.0 ± 2.7**  (0 - 16) |
| Laying impaired ^1^ | 3.1 ± 5.1  (0 - 32) | 1.7 ± 3.4  (0 - 18) | 2.7 ± 7.3 (0-46) | 2.2 ± 7.0  (0 - 46) | 0.9 ± 2.8  (0 - 12) | 0.2 ± 0.9  (0 - 6) |

^1^ excluding young flocks (< 24^th^ week of life) and molted flocks: MH, n = 155, FR, n = 51, NR, n = 47
MH: Mobile housing systems FR: stationary houses with free-range access; NR: stationary houses without free-range access
